# Phylogeny and Systematics of *Sassafras* (Lauraceae), an Interesting Genus with Disjunct Distributions in Eastern North America and East Asia

**DOI:** 10.3390/plants12061419

**Published:** 2023-03-22

**Authors:** Yunyan Zhang, Jingbo Zhou, David Y. P. Tng, Shuang Wang, Ying Wang, Ye Peng, Hong Liu, Zhongsheng Wang

**Affiliations:** 1 College of Life Sciences, Nanjing University, Nanjing 210023, China; zyynju@nju.edu.cn (Y.Z.);; 2 Centre for Rainforest Studies, The School for Field Studies, Yungaburra, Queensland 4884, Australia; 3 College of Biology and the Environment, Nanjing Forestry University, Nanjing 210037, China; 4 Center for Tropical Plant Conservation, Fairchild Tropical Botanic Garden, Coral Gables, Miami, FL 33156, USA; 5 Department of Earth and Environment, Florida International University, Miami, FL 33199, USA

**Keywords:** Lauraceae, *Sassafras*, inflorescence, involucre, molecular phylogeny, whole chloroplast genome

## Abstract

The Lauraceae is a family of the order Laurales, with 2500–3000 species comprising 50 genera, mainly distributed in tropical and subtropical evergreen broad-leaved forests. Up to two decades ago, the systematic classification of the Lauraceae was based on floral morphology, but molecular phylogenetic approaches have made tremendous advances in elucidating tribe- and genus-level relationships within the family in recent decades. Our review focused on the phylogeny and systematics of *Sassafras*, a genus of three species with highly disjunct distributions in eastern North America and East Asia, whose tribe affiliation within the Lauraceae has long been controversial. By synthesizing information on the floral biology and molecular phylogeny of *Sassafras*, this review aimed to explore the position of *Sassafras* within the Lauraceae, and to provide suggestions and implications for future phylogenetic studies. Our synthesis highlighted *Sassafras* as a transitional type between Cinnamomeae and Laureae with a closer genetic relationship with Cinnamomeae, as revealed by molecular phylogenetic evidence, while it shares many similar characteristics with Laureae in morphology. We therefore discovered that several molecular and morphological methods should be concurrently considered to illuminate the phylogeny and systematics of *Sassafras* in Lauraceae.

## 1. Introduction

The Lauraceae is a large and vital woody plant family (with the exception of the herbaceous parasite *Cassytha*), containing approximately 2500 to 3000 species from 50 genera. Species of Lauraceae are ecologically important in evergreen broad-leaved forests in tropical and subtropical regions [1], often forming a significant component of the taxonomic diversity of these forests. The first major taxonomic work on the Lauraceae was the *Systema Laurinarum* by the German botanist Nees von Esenbeck [2], which established 13 tribes within the family based on floral and fruit characters, such as involucral bract arrangement, inflorescence structure, the sexuality of the flowers, the number of anthers and fertile stamens, the presence/absence of cupules and the degree of cupule coating. Examining these features in greater detail, later studies established different classification systems for Lauraceae [3,4,5,6,7,8,9,10]. Among them, the Kostermans [6] and van der Werff and Richter [8] classification systems have played a significant role in identifying Lauraceae species, and have become the reference systems for the current systematic taxonomy of Lauraceae [11,12,13,14,15].

Concerning the Kostermans [6] and van der Werff and Richter [8] Lauraceae taxonomic systems, controversies have arisen regarding the systematic relationships within genera and the division of tribes. The dispute about the systematic position of *Sassafras* is of particular interest. Based on its inflorescence, which has no involucre, *Sassafras* was classified into the tribe Cinnamomeae within *Cinnamommum* by Kostermans [6]. In contrast, van der Werff and Richter [8] placed *Sassafras* within the tribe Laureae with the genera *Litsea* and *Lindera*. Although the tribe Laureae was characterized by umbellate inflorescence surrounded by involucral bracts, *Sassafras* is an exception in the Laureae sensu van der Werff and Richter [8], due to having a racemose inflorescence lacking involucral bracts. The above two taxonomic systems both emphasize the inflorescence type and the presence/absence of involucres in *Sassafras*, leading to *Sassafras*’s placement in different tribes.

With the aim of shedding light on the systematic placement of *Sassafras* within the Lauraceae, we reviewed the history of traditional taxonomic studies on the Lauraceae relevant to our genus of interest. In particular, we reviewed research pertaining to the main taxonomic characters used to delineate *Sassafras*, including floral morphology, inflorescence type and involucre peculiarities. Additionally, we synthesized this body of morphological literature with molecular phylogenetic work to identify gaps in our knowledge of the genus.

## 2. Natural History and Biogeography

The genus *Sassafras* consists of three extant species of deciduous trees and at least one or more extinct species. Members of the genus are known for their economic, medicinal and culinary properties [16,17]. The vegetative morphology of *Sassafras* is distinctive among the Lauraceae as their members possess varying leaf patterns on the same plant, including oval, bilobed (mitten-shaped), trilobed (three-pronged) and, on rare occasions, five-lobed leaves [18].

Czech botanist Jan Svatopluk Presl [19] formally described the genus based on *S. albidum* (Nutt.) Nees, an economically and medicinally useful species narrowly distributed in the east of North America and the most northernly occurring taxon of the Lauraceae [20]. The genus name is derived from the appellation “sassafras”, which was used by the botanist Nicholas Monardes centuries early to describe the same species in Florida. For over three-quarters of a century after the naming of *Sassafras albidum*, the taxon was considered a unique monotypic genus of the eastern North American flora. This north American species was first described by Linnaeus Carolus as *Laurus sassafras* [21], although Presl later moved this species from *Laurus* to the new genus *Sassafras* [19]. Nees [2] established the currently accepted name, *S. albidum*.

In 1891, British botanist William Hemsley described *Sassafras tzumu* (Hemsley) from middle eastern and southern China as *Lindera tzumu* Hemsl. according to the fruiting specimens and *Litsea laxiflora* Hemsl. based on the flowering specimens [22]. Later in 1907, Hemsley transferred *Sassafras tzumu* as the accepted name, treating *Litsea laxiflora* as its synonym [23]. Soon afterwards, Lecomte [24] observed that *S. tzumu* had bisexual flowers while *S. albidum* had unisexual flowers; he thus established a new genus, *Pseudosassafras* Lecomte, and renamed *S. tzumu* to *Pseudosassafras tzumu* (Hemsl.) Lecomte.

When it comes to the other Asian species from evergreen broad-leaved forests in central Taiwan, *Sassafras randaiense* was initially described as *Lindera randaiense* by Hayata in 1911 [25] and transferred by Rehder to *S. randaiense* (Hayata) Reher. later in 1920 [26]. However, according to the difference between 2-locular stamens and 4-locular stamens (one of the characteristics of *Sassafras*), in 1933, Kamikoti established a new genus, *Yushunia* Kamik., and renamed the two-celled *Sassafras randaiense* as *Yushunia randaiense* (Hayata) Kamik in 1933 [27].

Above all, the similarities/differences among the three *Sassafras* species led many botanists to assign them to a genus with three species, or the three species can be split as three monotypic genera, which made *Sassafras* an interesting case in taxonomy. The current disjunct distribution of extant *Sassafras* also highlights the well-known botanical affinities between eastern North America and east Asia, where *Sassafras* is one of three Lauraceae genera with such disjunct distributions, the other two being *Lindera* and *Litsea* [28,29].

*Sassafras albidum* was predicted to have diverged from its two siblings around 13.80 ± 2.29~16.69 ± 2.52 million years ago (mya); *S. randaiense* diverged from *S. tzumu* at about 0.61 ± 0.75~2.23 ± 0.76 mya, and *S. randaiense* in Taiwan was likely derived from *S. tzumu* from continental China [1]. A fourth extinct species, *Sassafras hesperia*, was described by Berry [30] from a fossil leaf from the Late Miocene in the Latah Formation sedimentary outcrops in eastern Washington and northwestern Idaho. Another fossil species with potential affinities with *Sassafras*, *Sassafrasoxylon gottwaldii*, was described by Poole et al. [31] from its woody fossils from Late Cretaceous (Santonian–Maastrichtian) sediments of the northern Antarctica Peninsula region, suggesting a potential Gondwanan origin of *Sassafras*, or at least its stem taxon. However, Nie et al. [1] pointed out that the ring-porous wood fossil of *Sassafrasoxylon gottwaldii* represents a stem taxon or another Lauraceae genus that formed growth rings in a seasonal and polar environment.

## 3. Floral Morphology and Monophyly of *Sassafras*

The three extant species of *Sassafras* differ primarily in their floral structure: *S. randaiense* varies from *S. tzumu* in terms of anthers, with the former’s anthers having two to four locules while the latter always has four locules. *S. albidum* is markedly distinct from its eastern Asian congeners because it lacks a pistillode and because of its unisexual flowers, as compared to the bisexual flowers of *S. tzumu* and *S. randaiense*. Additionally, *S. albidum* has six staminodes in the pistillate flowers and nine relatively well-developed stamens rather than the three staminodes found in the Asian species [1,26,32,33].

The type species *S. albidum* is described as being dioecious [26,29,34,35]. Likewise, *S. tzumu* was described as a dioecious species with very similar male and female flowers, [23] which are hermaphroditic [24,36] and polygamo-dioecious [37]. Rehder stated that the flowers of *S. tzumu* were androdioecious (with staminate or hermaphroditic flowers), and compared with fertile hermaphroditic flowers, the female function of the male flower was degraded (with a smaller and deformed ovary) but accidentally fertile [26]. *S. albidum* can therefore be distinguished from *S. tzumu* and *S. randaiense* by its more evolved dioecism (i.e., unisexual flowers vs bisexual flowers, more stamen degeneration or pistil loss).

*Sassafras randaiense* was first depicted as having staminate flowers with nine stamens and staminodia by Hayata, resembling that of *S. albidum* [25,26,38]. Later, Keng [39] stated that the differences between the staminate and pistillate flowers of the two Asian siblings are minor, with staminate flowers characterized by relatively larger androecia and smaller gynoecia in comparison to those of the pistillate flowers. A recent illuminating investigation on the floral morphology of *S. randaiense* was carried out by Chung et al. [33]. They demonstrated that the flower of the Taiwanese *Sassafras* is bisexual and its inflorescence is a determinate botryoid raceme comprising six tepals in two whorls, nine stamens in three whorls and three sagittate staminodia as the fourth whorl, with a central gynoecium [33]. Additionally, the sexual system of *S. randaiense* is possibly synchronously dichogamous (the species contains two flower morphologies that hinge on the timing of flower opening); in the female phase, the flowers of *S. randaiense* are usually described as hermaphroditic, while in the male stage, the wilted gynoecia are likely to be interpreted as nonfunctional [33]. In such an instance, *S. randaiense* is likely to be interpreted as a staminate individual [33], which we assumed to be a phenomenon of temporary dioecism. Keng [39] also proposed that “the male flowers had larger stamens and smaller pistils than the female flowers”, exposing the various flower morphological characteristics displayed at different sexual development stages.

Speaking of the other Asian species of *Sassafras*, *S. tzumu*, it was earlier documented as a dioecious tree [23]. Shen et al. [40] further proved that the flower of *S. tzumu* was bisexual through a large number of floral observations. Gu et al. [41] and Wang et al. [42] supported Shen et al.’s view to some extent, and suggested that *S. tzumu* has bisexual flowers with precocious pistils. Yang et al. [32] investigated the floral morphology of the species in detail and found that it has determinate botryoid racemes and possesses protogynous bisexual flowers similar to those of *S. randaiense*. They also observed its two phenological phases: in a flower, the pistil develops first, the stigma is fresh and white, stamens of the outer two whorls spread, the anthers do not open, and the staminodes secrete nectar at this stage. In the second phase, the stigma becomes brown, the staminodes are withered, the stamens of the third whorl stand up and surround the pistil, the glands of the third whorl of stamens secrete nectar, and the anthers open and release pollen [32].

Additionally, van der Werff and Richter [3] emphasized the importance of introrse or extrorse anthers of the third-whorl stamens of hermaphroditic laurel flowers in the taxonomy of Lauraceae at the generic level. Rohde et al. [43] stated that the third-whorl stamens are extrorse in some hermaphroditic flowers of Lauraceae, and the formation of this structure is caused by inner stamens that are upright and closed to the style at the male flowering stage, so there is no space allowing them to fold inward. Chung et al. [33] observed that the anthers of the first and second stamen whorl of *S. randaiense* are introrse, while the third whorl is apparently extrorse or latrorse (in the male phase, those of the third whorl are extrorse). Yang et al. [32] found that the bisexual flower of *S. tzumu* is protogynous, and the anthers of the first and second staminal whorl are introrse, like those of *S. randaiense*, while the opening of anther locules of the third staminal whorl is variable: the upper two locules are smaller, circular or nearly so, apical and/or slightly introrse, and the lower two locules are relatively bigger, ovate to elliptic, and latrorse, not introrse or extrorse, differing from the extrorse third-whorl anthers in *S. randaiense* [33]. Therefore, the third-whorl extrorse/introrse stamen is not a sufficiently stable morphological characteristic for the classification of Lauraceae at the generic level.

In spite of the differences in floral structure for the above three extant *Sassafras* species (*S. albidum*, *S. randaiense*, and *S. tzumu*), the majority of botanists and systematists follow Rehder’s classification [26], which merged these three species into a single genus. Nie at al.’s phylogenetic study [1] and many subsequent molecular systematic studies (we will review these in Section 5) also demonstrated the monophyly of these congeners.

## 4. Morphological Contentions over the Tribal Placement of *Sassafras*

### 4.1. To Involve the Involucre or Not?

The tribal classification of *Sassafras* has traditionally been based on floral and inflorescence morphology and has been a contentious issue for almost two centuries. This contention occurs against the backdrop of shifting opinions on the tribal boundaries of the Lauraceae in general.

According to the only diagnostic character of “folia decidua”, Nees von Esenbeck [2] first placed *Sassafras* with the genus *Benzoin* (*Lindera*) into the tribe Flaviflorae, which is synonymous with the tribe Laureae of van der Werff and Richter [3]. Contrary to Nees von Esenbeck’s [2] classification system, Meissner [10] suggested that *Sassafras* belongs in the tribe Oreodaphneae. Additionally, the tribe Litseeae in Kostermans’s system [6] is similar to the tribe Laureae as described by van der Werff and Richter [8], whose representative genera consisted of *Litsea* and *Lindera*, with flowers in pseudo-umbals surrounded by persistent, decussate large bracts. In terms of the involucre-enclosed inflorescences, *Sassafras* was classified into the tribe Cinnamomeae by Kostermans [6] with *Ocotea*, *Cinnamomum*, *Actinodaphne* and *Umbellularia*. However, *Sassafras* was placed in the tribe Laureae by van der Werff and Richter due to it showing racemose inflorescences surrounded by involucral bracts [8].

Clearly, whether *Sassafras* has involucres or not is at the root of the controversy regarding its classification. Kostermans [6] classified *Sassafras* in the tribe Cinnamomeae on the basis of its lack of an involucre, but also recognized that the genus has alternate and deciduous bracts that fall off before flowering. Moreover, Kostermans [6] also believed that the bracts of *Sassafras* had the same origin as those of *Litsea* and *Lindera*. Tsui [44,45] agreed with Kostermans [6], and suggested that the decussate involucre was the key character distinguishing the tribe Litseeae (Laureae) from the tribe Cinnamomeae. Rohwer [4] believed that the young inflorescences of *S. randaiense* were enclosed in vegetative winter buds by four to six reciprocal bracts. Additionally, Liao [46] suggested that these bracts formed involucres, but Chung et al. [33] thought that this view was distorted. Rohde et al. [43] also agreed that the involucre of *Sassafras* was different from that of species within the tribe Laureae, and suggested that most species of the tribe Laureae have pseudo-umbel axillary inflorescences surrounded by alternating bracts, and that the bracts remained after flowering, while in *S. albidum*, the bracts or transitional leaves subtending the inflorescences are persistent at anthesis [32]. Furthermore, *Sassafras* produced racemes from spirally arranged bud scales and axils of transitional leaves in early spring germination. It remains to be further studied whether the formation of inflorescences is related to the evolution of spirally inserted involucres to reciprocal involucres.

### 4.2. The Inflorescence and Anther Locule Number

The inflorescence type of *Sassafras* is the most important character for determining the tribe to which it belongs and studying phylogenetic relationships within its genera. Mez [8] ascribed *Sassafras* to Litseeae mainly on the basis of its racemes with bracts, while Bentham and Hooker classified it into the *Litsea* genus [9] due to its introrse and dense stamens and a short, subsessile inflorescence. Rehder [26] agreed with Mez’s [8] classification of *Sassafras*. Kostermans [6] described the flowers of *Sassafras* as shortened racemes (pseudo-umbals) surrounded by deciduous, alternate bract leaves. However, van der Werff and Richter [3] classified *Sassafras* into the tribe Laureae, and suggested that most tribes of Laureae species have a raceme with a bract at the base of the pedicel, an extremely shortened inflorescence axis and inflorescences that appear umbellate. Van der Werff [47] further suggested that the flowers of species of the tribe Laureae are arranged in umbels, with young inflorescences enclosed in decussate bracts. Additionally, the whole structure of the inflorescence resembles a raceme of umbels as the umbels were arranged along a leafless, short shoot. Chung et al. [33] discovered that the inflorescence of *S. randaiense* ends with a terminal flower. On average, 13 flowers of each inflorescence are arranged in a highly reduced panicle (61.3%) or raceme-like cyme (38.7%). These flowers are closely clustered around the terminal buds (pseudo-terminal) of cultivation, forming an appearance similar to the inflorescence type of the tribe Laureae. Yang et al. [32] found that the inflorescences of *S. tzumu* are developed from the large terminal perulate buds, and *S. tzumu* has raceme-like but determinate/botryoid inflorescences, while the inflorescences of *Lindera* and *Litsea* are usually umbellate. Each inflorescence of *S. tzumu* has approximately 11 pedicellate flowers, which can be arranged alternately, nearly oppositely or verticillately on the densely brownish pubescent peduncle.

The anther locule number is also an important taxonomic characteristic in the family of Lauraceae. Four locular anthers are believed to be the plesiomorphic feature of the Lauraceae, such as in the tribe Cinnamomeae, whereas two locular anthers are considered as an evolutionary trait that could have originated from four locular anthers multiple times [32,48]. As for the genus *Sassafras*, the number of anther locules varies from four (*S. tzumu*, *S. randaiense* and *S. albidum*) to two (*S. randaiense* and *S. albidum*) [26,32,33]. In *S. albidum*, as well as a few *Lindera* and *Ocotea* species, the two locular anthers originated from the reduction in the upper pair of locules [32,48]. Additionally, Yang et al. [32] indicated that the deciduous habit of *Sassafras* plants is considered to originate independently from *Litsea* and *Lindera*. They further concluded that the racemose inflorescence of ancestral *Sassafras* originated from the thyrsoid–cymose inflorescence in Cinnamomum, while the similarity of racemose inflorescences between *Sassafras* and some species of Laureae resulted from parallel evolution [32].

## 5. Molecular Phylogeny of *Sassafras*

### 5.1. Application of Single Gene or Polygene Fragment in Phylogeny of Sassafras

As molecular marker technology has become widely used in systematic research, Rohwer [11] employed the larger part of the *mat*K gene and the (3′) adjacent spacer sequence to construct the first phylogenetic tree of the Lauraceae, encompassing 48 species from 29 genera. In Rohwe’s [11] phylogenetic tree, the traditional subdivisions in former classification systems of the family (involucrate and non-involucrate inflorescences) were not supported, while Lauraceae species were divided into two groups considering their historical and geographical origins: the Gondwanan group and the Laurasian–South American group. *Sassafras* was clustered with *Neolitsea* but had poor support in the latter group.

Based on *trn*L-*trn*F, *trn*T-*trn*L, *psb*A-*trn*H and *rpl*16 of cpDNA, and the 5′ end of 26S rDNA, a phylogenetic tree of 122 species in 44 genera of Lauraceae was constructed by Chanderbali et al. [12]; the terminal branch was found radiating in Lauraceae from the early Eocene, and it was named the Perseeae–Laureae clade, which mainly included the *Persea* group, Laureae, Cinnamomeae and the *Ocotea* complex group. Almost all genera with controversial phylogenetic relationships belonged to this Perseeae–Laureae clade. In Chanderbali et al. [12], the sister relationship between *Sassafras* and Laureae was moderately supported by the topological constraint of the monophyly of the Laureae. Without the topological constraint, *Sassafras* became sister to the Cinnamomeae, which was composed of the *Ocotea* complex group and the *Cinnamomum* group with intermediate support. Rohwer and Rudolp [49] re-examined the phylogeny of 48 species of 30 genera of Lauraceae with the data sets of *trn*K, *mat*K and *trn*K introns. The phylogenetic tree [49] was still a terminal branch including the *Persea* group and Laureae–Cinnamomeae clade, and the terminal branch was described as the core Lauraceae group. Within the core Lauraceae group, almost all the genera (including *Sassafras* with moderate support) belonged to the Laureae–Cinnamomeae branch, except the *Persea* group, which has been recognized in previous molecular studies [11,12]. Among the above-mentioned earlier phylogenetic studies, *Sassafras* was classified into the terminal Laureae–Cinnamomeae branch, which suggested *Sassafras* had different degrees of similarity with Laureae or Cinnamomum; however, these early studies failed to confirm the accurate phylogenetic position of *Sassafras*.

Based on these combined data (*mat*K of cpDNA and ribosomal ITS sequence), Li et al. [13] carried out a phylogenetic analysis of representative groups of Laureae (23 species of 11 genera); the results supported *Sassafras*’s classification as Cinnamomeae, and that Cinnamomeae was sister to Laureae. Nie et al. [1] studied the phylogenetic relationships of 48 species from 29 genera of Lauraceae using a ribosomal ITS sequence and three non-coding regions of cpDNA (*rpl*16, *trn*L-F, and *psb*A-*trn*H); the research strongly supported that *Sassafras* belonged to the Cinnamomeae branch and that it diverged from other taxa of Cinnamomeae at about 33.02 ± 2.00 mya. Rohde et al. [39] carried out a more detailed phylogenetic study according to ITS, *psb*A-*trn*H and *trn*G-*trn*S sequences; within ITS data, *Sassafras* was sister to the *Cinnamommum* sect. *Cinnamum* and Laureae with moderate support, while in the phylogenetic tree of *psb*A-*trn*H and *trn*G-*trn*S sequences, the sister relationship between *Sassafras* and the *Cinnamomum* sect. *Camphora* (the core section of Cinnamomeae) was moderately supported. However, the combined data of gene fragments from the nucleus and chloroplast still indicate the controversial nature of the phylogenetic relationship of *Sassafras* with Lauraceae.

### 5.2. Insights from Whole Chloroplast Genome Sequencing

Compared with gene fragment data, the whole chloroplast genome contained more abundant and valuable genetic information on plants [14,43]. Song et al. [14] constructed a molecular phylogenetic tree based on the chloroplast genome of 44 Magnoliids (including 15 new Lauraceae species and 19 published Lauraceae data); within this tree, Laureae and Cinnamomeae were completely separated (ML-BS = 100%), and *Sassafras* was strongly predicated to be clustered with Cinnamomeae (ML-BS = 100%). Zhao et al. [50] systematically analyzed chloroplast genome data from 30 species of the Perseeae–Laureae clade of Lauraceae (including nine new chloroplast genome data of *Lindera*), selecting two *Endiandra* species as an outgroup; the topological structure of the phylogenetic tree showed three major branches: the *Persea–Machilus* clade, the *Ocotea–Cinnamomum* clade and the Laureae clade. *Sassafras* was classified into the *Ocotea–Cinnamomum* clade, which was sister to the Laureae clade. Liao et al. [51] sequenced the whole chloroplast genome of *Parasassafras conferflorum* (Meisn.) D.G Long and reconstructed the phylogenetic relationship of 28 species of the Perseeae–Laureae clade using complete chloroplast sequences, with two *Endiandra* species as an outgroup, resulting in three major branches similar to those found by Zhao et al. [50], and also found *Sassafras* belonged to the *Ocotea–Cinnamomum* clade. Jo et al. [52] analyzed the phylogeny of 49 Lauraceae species (including data on 20 new complete plastomes) using 77 protein coding sequences and four rRNA genes of whole chloroplast genome sequences, obtaining six clear branches: Cryptocaryeae, *Neocinnamomum*, *Caryodaphnopsis,* Perseeae, Cinnamomeae and Laureae (*Sassafras* was classified into Cinnamomeae with *Cinnamomum*). Song et al. [15] further rebuilt the phylogenetic relationships of 120 whole chloroplast genomes of Lauraceae and related species, and their results show Lauraceae is monophyletic with nine highly supported clades (*Hypodaphnis* clade, *Beilschmiedia–Cryptocarya* clade, *Cassytha* clade, *Neocinnamomum* clade, *Caryodaphnopsis* clade, *Chlorocardium–Mezilaurus* clade, *Machilus–Persea* clade, *Cinnamomum–Ocotea* clade, and *Laurus–Neolitsea* clade); *Sassafras* was clustered with *Cinnamomum* in the clade *Cinnamomum–Ocotea*.

In conclusion, the majority of phylogenetic research on Lauraceae based on whole chloroplast genome data supports the establishment of Cinnamomeae and that *Sassafras* belongs to Cinnamomeae; the above genetic relationship between Cinnamomum and Laureae is widely accepted.

## 6. Discussion

Since Nees [2] established the taxonomic hierarchy of “Tribus” (Lauraceae), there have been two viewpoints on the controversy of *Sassafras*’s tribe affiliation: one group suggested that *Sassafras* belonged to the raceme–pseudo-umbel group, and classified *Sassafras* into the tribe Flaviflorae [2], tribe Litseeae [8] or tribe Laureae [3] with pseudo-umbel and involucre species such as *Lindera* and *Litsea*; the other group asserted that *Sassafras* was included in the raceme–cymose panicle group, and classified *Sassafras* into the tribe Oreodophneae [10] or tribe Cinnamomeae [6] with *Cinnamomum* and *Ocotea*, which had the common morphological characteristics of a cymose panicle without a involucre. The key focus of the above controversies was the inflorescence type (raceme, botryoid and capitellate inflorescence composed of raceme inflorescence) of *Sassafras*, and the close evolutionary relationship between this undetermined inflorescence type and pseudo-umbel inflorescence, cyme, panicle and inflorescence characteristics.

The shortened branchlets and brachyblast type suggested by Li [53] and Tsui [44] were the most representative evolutionary series of the inflorescence. Tsui [44,53] considered that the whole shortened branchlets of *Lindera* and *Sassafras* were surrounded by leathery bracts (forming mixed buds). Considering the similar appearance of *Lindera* (especially Sect. Palminervia Meissn.) and *Sassafras*, Tsui [44,45] believed the inflorescence of *Lindera* might have been a simplified form of the inflorescence of *Sassafras-*like ancestors. Additionally, in terms of shortened branchlets, an inflorescence evolutionary pattern that is supported by an increasing amount of research [4,13] was suggested by Tsui [44,45], showing an extremely shortened raceme axis shaped like an umbel [44,53]. Paleobotanical evidence also indicated that *Sassafras* had been found in northern Asia and North America during the early Cretaceous, while *Lindera* and *Litsea* were not found until the beginning of the Tertiary (*Writing Group of Cenozoic Plants of China*, 1978). Hence, the *Lindera* group could originate from ancestors similar to those of *Sassafras* [44]. Additionally, Chung et al. [31] found that some individual florets within the raceme of *S. randaiense* remained lateral flowers (the flowers of *S. randaiense* are arranged in a raceme-like cyme (38.7%)) or had a highly reduced panicle (61.3%), which could be regarded as the plesiomorph of a simplified cyme, panicle and inflorescence). In Lauraceae, the raceme inflorescence simplified to a single floret, further providing strong evidence for the inference that the cymose panicle was highly simplified to a raceme-like inflorescence similar to that of *Sassafras.* In summary, the pattern of inflorescence evolution in Lauraceae can be deduced as follows: the cymose panicle was highly simplified to a raceme-like inflorescence, and then the inflorescence axis was shortened to an umbel-like inflorescence. Therefore, *Sassafras* with raceme inflorescences represents a transitional taxon during the evolution of Lauraceae, which has been and always will be an important viewpoint in the phylogenetic study of Lauraceae.

Van der Werff and Richter [3] dismantled the tribe Cinnamomeae and established the tribe Laureae on the basis of dioecism and introrse inner anthers. However, *Sassafras* is not a simple dioecious genus. Flowers of *S. tzumu* are structurally hermaphroditic (but functionally unisexual); *S. randaiense*’s pistils ripen first, and it was described as a bisexual flower during pistil ripening, becoming a unisexual flower with a withered pistil at the male stage, and *S. albidum* is dioecious in terms of stamen degeneration or pistil loss. Thus, *Sassafras* could not be classified into the tribe Laureae based on dioecism. Moreover, Chung et al. [33] suggested that the introrse inner anthers of *S. randaiense* varied at different sexual development stages, and could not be regarded as a relatively stable taxonomic trait to classify *Sassafras* into the tribe Laureae.

In previous phylogenetic studies of Lauraceae based on gene fragments, representative genera, such as *Sassafras*, *Litsea*, *Lindera*, *Neolitsea*, *Actinodaphne*, *Cinnamomum*, *Ocotea* and *Umbellularia*, were found to form a terminal branch of the phylogenetic tree [11,49], and are known as the core Lauraceae group [47]. However, because of the low genetic divergence and phylogenetic information, the circumscription of Lauraceae and Cinnamomeae cannot be clearly clarified, and *Sassafras* represented an ambiguous phylogenetic position (*Cinnamomum–Ocotea* complex or Lauraceae) [1,11,49]. With the application of the whole chloroplast genome in phylogenetic research, many targeted or representative molecular results for Lauraceae were found to support the establishment of Cinnamomeae, *Sassafras*’s status as a member of Cinnamomeae and the status of Cinnamomeae as sister to Laureae [14,50,51,52]. In conclusion, the plastid data revealed that the genus *Sassafras* had closer genetic relationship with and belonged to the tribe Cinnamomeae [14,15,50,51,52], while nuclear phylogeny [1,11,49] showed *Sassafras* was a sister to the genus *Lindera* (Laureae). This phenomenon of “cytonuclear conflict” could be explained by the ancient hybridization/introgression or incomplete lineage sorting.

Although detailed morphological studies, such as those on embryos and anthers, do not support *Sassafras* as a member of Laureae, and molecular phylogenetic research tends to classify *Sassafras* into Cinnamomeae, it is still necessary to combine the morphological characters and molecular data to illuminate the precise phylogenetic relationship of *Sassafras*. Chung et al. [33] provided evidence supporting the simplification of the raceme of S. randaiense derived from a cymose panicle. Yang et al. [32] found *S. tzumu* had determinate botryoid racemes, but they pointed out that the racemose inflorescence of ancestral *Sassafras* originated from the thyrsoid–cymose inflorescence in Cinnamomum, and the similarity of racemose inflorescences between *Sassafras* and some members of Laureae was discovered to originate from parallel evolution. More detailed observation and statistical work should be carried out for *S. albidum* in the future to explore the relationship between the raceme of *Sassafras* and cymose panicle. We do believe traditional structural botany methods will be valuable to find more direct plesiomorphic features, providing morphology and structural anatomy evidence of *Sassafras* flower to inflorescence evolution of Lauraceae. Additionally, considering *Sassafras* clustered to the *Cinnamomum–Ocotea* complex in the whole chloroplast phylogenetic tree [14,15,50,51,52], the observation and statistics of flower morphological characteristics to Cinnamomeae (e.g., *Ocotea*) also need to be carried out to verify whether the cymose panicle (especially lateral cymes) existed (e.g., *Cinnamomum*, *Ocotea*) and whether this phenomenon was simplified or not.

A focus on selecting DNA barcodes (combinations) with high genetic divergence, expanding the sampling range (some gene fragments vary greatly within species, which will lead to differences in the phylogenetic tree branch) and systematically elucidating the genetic evolutionary relationship of *Sassafras* will be key in future research on Lauraceae. However, merely relying on the molecular data will likely lead to random and systematic errors, thus making it challenging to differentiate between the phylogenetic tree and real evolutionary relationships. Therefore, we also believe that, via the combination of both molecular and morphological data, a more convincing phylogenetic tree can be established. Additionally, whole-genome data [54,55] and inflorescence-related genes [55] revealed by genomic data will illuminate the inflorescence evolutionary path and phylogenetic relationship of Lauraceae.

## Data Availability

No new data were created or analyzed in this study. Data sharing is not applicable to this article.
